# Population Dynamics Analysis of *Chromochloris zofingiensis*: A Flow-Cytometry-Based Approach

**DOI:** 10.3390/plants15050724

**Published:** 2026-02-27

**Authors:** Yob Ihadjadene, Alina Wulff, Thomas Walther, Stefan Streif, Felix Krujatz

**Affiliations:** 1 Professorship Automatic Control & System Dynamics, Chemnitz University of Technology, 09126 Chemnitz, Germany; yob.ihadjadene@etit.tu-chemnitz.de (Y.I.); stefan.streif@etit.tu-chemnitz.de (S.S.); 2 Institute of Natural Materials Technology, Dresden University of Technology, 01069 Dresden, Germany; 3 biotopa gGmbH—Center for Applied Aquaculture & Bioeconomy, 01454 Radeberg, Germany; 4 Department of Biotechnology, Technische Hochschule Mannheim, 68163 Mannheim, Germany; 5 Fraunhofer Institute for Molecular Biology and Applied Ecology, Department of Bioresources, 35392 Giessen, Germany

**Keywords:** population dynamics, *Chromochloris zofingiensis*, flow cytometry, microalgae, multiple fission

## Abstract

The design and optimization of microalgae processes are usually focused on maximizing biomass productivity, neglecting the impact of cell-to-cell heterogeneity. Flow cytometry (FCM) represents a powerful and high-throughput tool for analyzing and examining microalgae intrinsic characteristics, such as their physiology, metabolism and response at the single-cell level. The aim of this work is to develop a novel FCM sensor-based single-cell analysis method to monitor and study the effect of several process conditions, mainly variations of light spectral composition (blue, red and green), nitrogen depletion and moderate osmotic stress conditions (0.2 M NaCl), on the subpopulation structure and dynamics of the green microalgae *Chromochloris zofingiensis*, a natural source for lipids, proteins and carotenoids. The FCM procedures developed in this study proved to be effective for monitoring the population dynamics of microalgae, demonstrating how the process conditions have a direct and significant impact on population heterogeneity of *C. zofingiensis* on a single-cell level. Cell division was found to be adversely affected by the moderate osmotic stress (N^+^S^+^), nitrogen depletion (N^−^), and their combined occurrence (N^−^S^+^), independent of the light spectral composition used for culture illumination. In terms of cell-to-cell heterogeneity, a higher proportion of large cells (~20 µm) was observed under green light across all conditions with 21%, 29%, 35% and 52% under N^−^, N^−^S^+^, N^+^S^+^ and N^+^ conditions, respectively, followed by red light combined with osmotic stress (46%), whereas blue light consistently led to a predominance of smaller cells (≤4 µm) with 30%, 47%, 50% and 55% under N^+^S^+^, N^+^, N^−^S^+^ and N^−^ conditions, respectively.

## 1. Introduction

Microalgae are gaining importance in the bioeconomy and biotechnology sectors as a sustainable and promising source of natural high-value bioactive compounds [1,2]. This includes pigments, proteins, vitamins, carbohydrates and polyunsaturated fatty acids (PUFAs), with wide applications in food, feed, nutraceuticals, pharmaceuticals and cosmetics [3,4,5,6,7,8,9,10,11].

Developing and optimizing microalgal cultivation requires a comprehensive understanding of the cells’ intrinsic physiological characteristics and their dynamic responses to critical process parameters, including both qualitative (spectral composition) and quantitative (intensity) aspects of light, nutrient availability, temperature, pH and salinity, all of which profoundly impact their growth kinetics and metabolism [2,12]. However, identifying these characteristics usually involves labor-intensive and time-consuming analytical procedures [13], which highlights the urgent need for rapid and reliable alternative approaches.

A critical yet often-underestimated aspect of microalgal bioprocesses is the population heterogeneity, arising from technical and cellular reasons at the macro- and microscale, respectively. Heterogeneity at the level of cell populations is primarily driven by the non-uniform process conditions in the photobioreactor (PBR) environment [14]. Photoautotrophic bioprocesses are particularly distinctive in this context, as they exhibit two additional dimensions of process complexity compared to conventional heterotrophic fermentations: the spatially uneven distribution of photosynthetically active radiation (PAR) in PBRs and the heterogeneous mass transfer of carbon dioxide (CO_2_) into the liquid phase [15]. The pronounced technical heterogeneity within PBRs, together with various extrinsic and intrinsic factors, gives rise to substantial phenotypic heterogeneity on the cell population level, indicated by a variability in metabolic activity [16].

A comprehensive understanding of the cellular population structure is therefore essential to ensure reproducible and efficient operation of phototrophic bioprocesses since the physiological state of the cells directly impacts both upstream processing—such as inoculum quality and growth performance—and downstream processing, including harvesting and extraction efficiency, which are strongly affected by the cell size distribution and effective biovolume. Usai et al. [17] demonstrated that the biovolume of microalgal cells may vary by a factor of 2–15 depending on their physiological state, with a 2.5-fold increase in cell diameter corresponding to a 16-fold increase in cellular biovolume.

Among the analytical techniques used to characterize cellular physiology at a cellular level, flow cytometry (FCM) stands out for its high-throughput capacity to analyze cellular parameters such as cell size, granularity, storage compounds, and viability across a broad range of microorganisms, including microalgae [12,13,18,19,20,21,22].

*Chromochloris zofingiensis* is a unicellular freshwater microalga that has attracted considerable interest in biotechnology as a promising natural source of high-value carotenoids, particularly astaxanthin, canthaxanthin, β-carotene and lutein [23,24,25,26,27]. This species propagates through an asexual multiple-fission cell cycle, during which DNA replication and nuclear division occur multiple times prior to cytokinesis [28]. Consequently, polynuclear cells (PNCs), also referred to as mother cells, can be observed, where the number of daughter cells formed within a mother cell is determined by the frequency of DNA replication and nuclear division events preceding cell cleavage [29,30]. As *C. zofingiensis* progresses through its cell cycle, individual cells undergo growth, commitment to division, DNA replication, and nuclear division, thereby generating PNC subpopulations. Each subpopulation is characterized by the production of 2^n^ daughter cells from a single mother cell, where the exponent n represents the number of DNA replication cycles completed and reflects the degree of heterogeneity within the population [28,30]. While the majority of studies on *Chromochloris zofingiensis* have primarily focused on optimizing biomass productivity and enhancing astaxanthin and lipid biosynthesis under diverse cultivation conditions [23,31,32,33,34,35,36,37,38], the impact of these process parameters on cell-to-cell heterogeneity and population dynamics within *C. zofingiensis* cultures has remained largely unexplored.

In this context, the objective of the present study is to develop a novel quantitative flow cytometry-based single-cell analysis technique that advances beyond conventional microalgal FCM studies focused on bulk-averaged signals. The proposed approach is used to investigate and monitor the population dynamics and cell-to-cell heterogeneity of *Chromochloris zofingiensis* in response to variations in process conditions—namely nitrogen availability, osmotic stress by moderate salinity (0.2 M NaCl), and their combined effect—under three distinct light spectral compositions (red, blue, and green). By resolving stress- and light-induced phenotypic shifts as changes in multivariate population distributions, this study provides an integrated perspective that has not been previously reported in the literature.

## 2. Results and Discussion

### 2.1. Cell Population Growth Dynamics and Division

**Figure 1:**
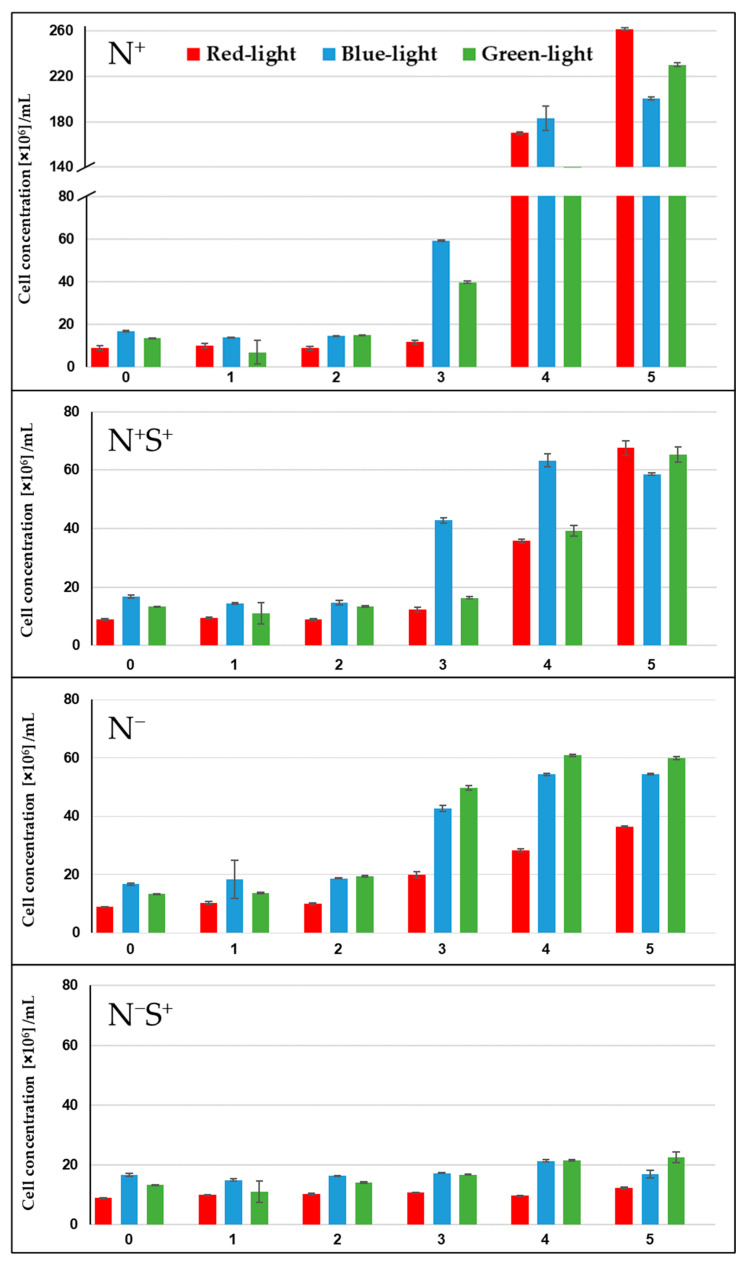
Cell concentration [cells × 10^6^/mL] over the cultivation time [days]. The colors of the bars correspond to the light colors (spectrum) during cultivation (red, blue, and green at 150 µmol∙m^−2^∙s^−1^). The data are expressed as mean ± SD (n = 3). N^+^ (control); N^−^ (nitrate depleted); N^+^S^+^ (moderate salinity); N^−^S^+^ (nitrate depleted and moderate salinity).

Accurate quantification of the physiological state of microalgal cells is essential for assessing their responses to changing process conditions [12]. Here, FCM was employed as a high-throughput technique to characterize the subpopulation dynamics of *Chromochloris zofingiensis*. To enable a quantitative interpretation, the forward light scattering (FSC) signals were calibrated using polystyrene beads of known particle size, thereby allowing precise assessment of the physiological responses of *C. zofingiensis*—including cell division behavior and cell size dynamics—as illustrated in Figure 1.

Across all tested conditions in the high-density (HD) cultivation system, an adaptation phase of approximately two days was observed before *C. zofingiensis* cells initiated cell division. Under the control condition (N^+^), the cultures reached the highest cell concentrations, exceeding 180 × 10^6^ cells/mL after five days of HD cultivation across all light spectral compositions. This was followed by the N^+^S^+^ condition (>60 × 10^6^ cells/mL), the N^−^ condition (40–60 × 10^6^ cells/mL), and finally the N^−^S^+^ condition (10–20 × 10^6^ cells/mL). These results indicate that the combined effect of nitrogen depletion and moderate osmotic stress exerted the highest impact on *C. zofingiensis* physiology, substantially impairing cell division capacity. Among the nitrogen-sufficient treatments (N^+^ and N^+^S^+^), cultures exposed to red light consistently exhibited the highest cell concentrations by day 5, followed by those under green and blue light, respectively.

These findings contrast with those reported by Ugwu et al. [39] for *C. zofingiensis* (strain No. 2175, National Institute for Environmental Studies, Tsukuba, Japan), who observed higher specific growth rates under blue light, followed by red light, across phototrophic, heterotrophic, and mixotrophic cultivation modes. The apparent discrepancy can likely be attributed to methodological differences of the studies, particularly the use of absorbance at 660 nm as a measure of cell growth. Since chlorophyll a exhibits a strong absorption peak at approximately 660 nm and chlorophyll b absorbs in the 630–675 nm spectral range [40,41], consequently, optical density measurement at this wavelength range is biased by the cellular pigment content. In contrast, the present results align with several previous studies identifying red light as the most favorable spectral range for promoting microalgal growth, including *Chlorella vulgaris* [41], *Arthrospira platensis* (formerly known as *Spirulina platensis*) [42], and *Botryococcus braunii* [43]. This observation is consistent with the fundamental photophysiology of most microalgal species, in which chlorophylls—the predominant photosynthetic pigments—exhibit significant absorption within the red and blue spectral range, thereby facilitating more efficient photochemistry and cellular energy level [40,41].

Under nitrogen-depleted conditions (N^−^ and N^−^S^+^), cultures exposed to green light exhibited the highest cell concentrations, followed by those cultivated under blue and red light, respectively. This trend is attributed to stress-induced metabolic shifts that favor carotenoids accumulation at the expense of chlorophyll synthesis. Similar observations were reported by Chen et al. [44], who demonstrated that the combination of blue light and nitrogen depletion enhanced astaxanthin accumulation relative to chlorophyll production in *C. zofingiensis* (strain ATCC 30412) compared to white light. However, their study also noted that excessive blue light intensity could inhibit both cell growth and astaxanthin biosynthesis, highlighting the sensitive balance between stress induction and metabolic productivity.

In *Chromochloris zofingiensis*, the main carotenoids reported under stress conditions are astaxanthin, canthaxanthin and adonixanthin [24,25,26]. While these carotenoids predominantly absorb light in the blue–green spectral region (~480–530 nm) [41], they are not functionally coupled to the photosynthetic apparatus, since their accumulation occurs in cytoplasmic lipid droplets outside the chloroplast [26].

The results also highlighted that increasing the osmotic stress of the medium (N^+^S^+^) through moderate salinity had a smaller impact on the physiology of *C. zofingiensis* than nitrate depletion, regardless of the light spectral composition used for illumination. This is evidenced by the N^+^S^+^ cultures, which consistently achieved higher cell concentrations than the N^−^ cultures across all spectral light conditions. These findings are consistent with the results reported by Mao et al. [45], who demonstrated that moderate salinity (0.2 M NaCl) can promote astaxanthin accumulation in *C. zofingiensis* without impairing cell growth or photosynthetic activity. In contrast, nitrogen depletion was shown to cause an immediate inhibition of photosynthesis and metabolic processes [46].

Similarly, the pronounced reduction in cell division capacity observed under the combined N^−^S^+^ stress condition supports the findings of previous studies indicating that *C. zofingiensis* physiology is strongly constrained by stress factors in a concentration-dependent manner—that is, higher salinity levels or lower nitrogen availability lead to reduced cell division and growth rates [23,45,46].

### 2.2. From Qualitative to Quantitative Cell Size Determination

The fundamental principle underlying FSC measurements in FCM is that the intensity of scattered light is directly influenced by the size of the cell when it passes through the laser beam [47]. Accordingly, the FSC signal is proportional to cell size—larger cells produce higher FSC values, whereas smaller cells yield lower signals. However, FSC values are inherently qualitative, as they are expressed in arbitrary units rather than absolute physical dimensions (µm). This poses a limitation for accurately estimating microalgal cell size, since FSC primarily reflects the relative optical cross-section of the cell rather than its true diameter.

To overcome this limitation, a calibration curve was established using a suspension of spherical polystyrene microbeads with defined diameters of 1 µm, 3 µm, and 10 µm. This calibration enabled the conversion of FSC signals into standardized size units, thereby allowing quantitative estimation of *C. zofingiensis* cell diameter with high accuracy (R^2^ = 0.997), as described by Equation (1):

(1) $$y=0.818\times {exp}^{{2.55\times 10}^{-4}x}$$

where *y* is the diameter of algae cells in (µm) and *x* is their corresponding FSC value.

Although FSC-based size calibration using spherical beads provides a robust approach for estimating relative cell size distributions, several inherent limitations must be considered particularly due to the morphological variability of microalgae species, since elongated, irregularly shaped, or polynuclear cells may scatter light differently [12], leading to deviations from bead-based calibration assumptions. Consequently, no universal correlation exists between FSC signal and cell size, as scattering also depends on instrument-specific optical properties and particle refractive index, requiring calibration-specific adjustments [12,48].

Several alternative single-cell techniques can address these limitations. Conventional imaging microscopy enables direct measurement of cell size and morphology, but is limited by low throughput and increased analysis effort [12,49,50]. Other light-microscopy–based approaches, such as flow imaging microscopy, offer label-free morphological information but are generally less suited for high-throughput population-level analyses compared to FCM [51]. Imaging flow cytometry combines FCM with image-based morphological analysis, allowing direct assessment of cell shape, size and division state, thereby reducing ambiguities associated with FSC-derived estimates for non-spherical or dividing cells, however, this technology typically involves higher instrumental complexity and computational demands [50,52,53].

### 2.3. From Cell Diameter to Cell Biovolume: Improving Resolution in Population Dynamics

**Figure 2:**
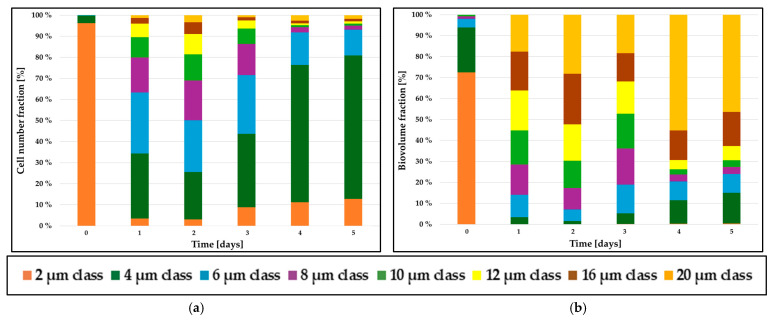
Comparative diagram of the temporal evolution of *C. zofingiensis* subpopulation composition expressed as the relative cell abundance of each diameter class within the total cell number (**a**) and the relative cell abundance of each diameter class within the total biovolume (**b**), for the N^+^S^+^ condition under red light illumination. In both panels, values are normalized to the total cell number or total biovolume, respectively, and diameter classes correspond to discrete size intervals defined in Section 3.6.

Microscopic analyses confirmed that *C. zofingiensis* cells exhibit a spherical morphology [27,28]. Consequently, their cellular biovolume can be derived from the calibrated FSC data, as described by Equations (2) and (3) in Section 3.6. The subpopulation structure in *C. zofingiensis* cultures can thus be expressed either in terms of cellular diameter or as active biovolume. Figure 2 presents a comparative visualization of these two-distribution metrics, highlighting the interpretative differences that emerge when transitioning from size-based to biovolumetric representation of the population structure.

In the diameter-based subpopulation analysis, small cells (<4 µm) numerically dominated the overall cell population. On day 0, cells with an average diameter of 2 µm accounted for more than 95% of the total cell count (Figure 2a). However, when the same population was evaluated in terms of biovolume, the relative contribution of these small cells decreased to approximately 70% (Figure 2b). This divergence between numerical and biovolumetric distributions became progressively more pronounced over the time of cultivation. By day 4, the disparity reached its maximum, as summarized in Table 1: cells with a diameter of 20 µm represented only ~2.5% of the total cell number, yet contributed disproportionately—approximately 55%—to the overall biovolume.

This shift arises from the cubic relationship between cell diameter and biovolume, whereby even a small fraction of large cells, e.g., 16 µm and 20 µm showed a substantial impact on biovolumetric parameters despite their low numerical abundance.

Thus, the diameter-based representation primarily reflected the numerical frequency of distinct size classes, whereas the biovolume-based representation emphasized the relative contribution of larger cells to the total biovolume. These two analytical perspectives could yield markedly different biological interpretations. Diameter-based analyses are particularly suitable for monitoring cell proliferation and division dynamics, as demonstrated in the study of the consecutive multiple-fission cell cycle of *C. zofingiensis* by Koren et al. [28].

Moreover, evaluating diameter-based cell size distributions provides essential insights for the optimization of downstream processing steps, including harvesting and product recovery. During harvesting, for instance, the selection and fine-tuning of operational parameters are closely dependent on an accurate understanding of cell size distribution, which directly influences separation efficiency and process performance [54,55,56,57].

In contrast, a biovolume-based analysis provides deeper insights into biomass accumulation, nutrient demand, and overall productivity within the culture. Numerous studies have demonstrated that cellular carbon, nitrogen, and phosphorus contents scale more closely with cellular biovolume than with linear dimensions such as cell diameter across a wide range of microalgal species, from spherical forms such as *Skeletonema marinoi*, *Prorocentrum micans*, *Thalassiosira pseudonana*, *Planktoniella sol*, and *Heterocapsa triquetra*, to more morphologically complex cells such as *Ceratium lineatum* [58,59,60]. Beyond diatoms and dinoflagellates, recent studies on green microalgae further support the relevance of biovolume-based metrics, showing that volume-derived descriptors better reflect biomass accumulation, physiological state, and stress responses than cell number or diameter alone in chlorophytes species such as *Chlorella vulgaris*, *Tetradesmus obliquus* (formerly known as *Scenedesmus obliquus*), *Desmodesmus* spp., *Chlamydomonas reinhardtii*, and *Coelastrella rubescens* [51,61,62].

Cell biovolume has also been shown to be a reliable predictor of the macromolecular biochemical metabolites, including protein, lipid, and carbohydrate levels, in various microalgal species such as *Auxenochlorella protothecoides* (formerly known as *Chlorella protothecoides*) and *A. pyrenoidosa* (formerly known as *Chlorella pyrenoidosa*) [63]. These findings underscore the value of biovolume-based metrics for linking cell morphology to biochemical composition, reinforcing their relevance for predicting culture productivity and guiding the optimization of bioprocess conditions.

### 2.4. Effect of Process Conditions on the Subpopulations Dynamics and Structure

**Figure 3:**
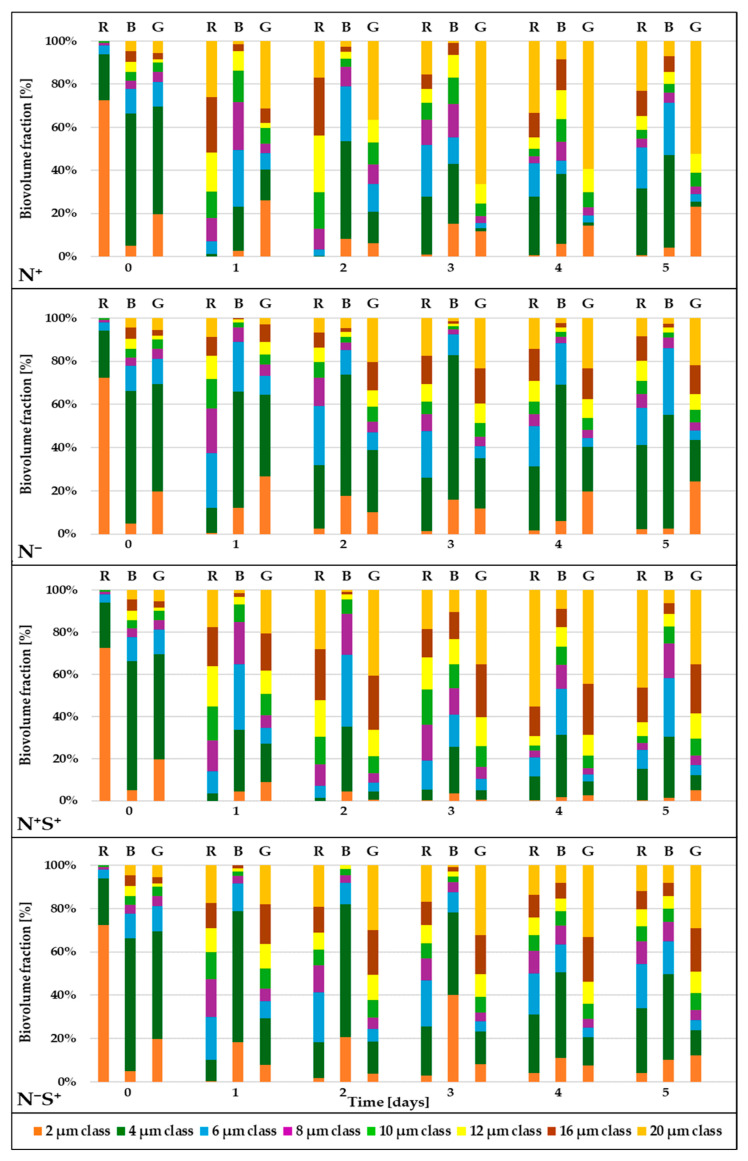
Temporal evolution of the relative biovolumetric distribution of *C. zofingiensis* cell diameter classes. Colors indicate distinct cell diameter classes, with the relative biovolume fraction (%) of each class normalized to the total population biovolume (y-axis). Light conditions are denoted by R (red), B (blue), and G (green). Nutrient and osmotic stress conditions are defined as follows: N^+^ (control); N^−^ (nitrate depleted); N^+^S^+^ (moderate salinity); N^−^S^+^ (nitrate depleted and moderate salinity).

The FCM routines developed in this study allowed for detailed analysis of *C. zofingiensis* subpopulation dynamics, providing insights into their relative proportions in terms of both cell number and biovolume. These routines enabled the differentiation of cells with diameters ranging from 2 to 20 µm and the corresponding estimation of their biovolume, facilitating precise characterization of subpopulation distributions within a single sample. This approach also allowed for the assessment of the impact of specific culture conditions on the cell size composition. Figure 3 illustrates the temporal evolution of *C. zofingiensis* subpopulation composition and dynamics over a 5-day cultivation period.

Although all *C. zofingiensis* cultures were initiated with approximately the same cell concentration on day 0 (Figure 1), their biovolumetric distributions were markedly different (Figure 3). Notably, most previous studies have characterized microalgal subpopulation composition based on relative cell abundance by diameter [28,49,64]. In contrast, the present study evaluates relative abundance by cellular biovolume, thereby highlighting the contribution of each subpopulation to the total biovolume and providing a more functionally relevant perspective on the subpopulation structure and dynamics.

The dynamics of cell growth and division during cultivation time are clearly reflected in the biovolumetric distribution of *C. zofingiensis*. Larger cells (>12 µm) initially increase in biovolume before undergoing division, which subsequently decreased their biovolumetric proportion while increasing the proportion of smaller cells (≤4 µm). This pattern is particularly pronounced under green light in the N^+^ condition: from day 0 to day 3, 20 µm cells expanded in biovolume, raising their biovolumetric fraction to 66%, followed by a decline to 52% by day 5 after division. Concurrently, the biovolumetric fraction of 2 µm cells increased from 12% on day 3 to 23% on day 5, reflecting the production of daughter cells from larger cells.

Similar trends have been observed by Pahija and Hui [64] in *Chlorococcum* sp., where small cells (<5 µm) tend to grow rapidly before dividing, whereas larger cells (>7 µm) exhibited slower growth and preferentially divide rather than increase in size. When considering cell counts, however, large *C. zofingiensis* cells (>12 µm) remain a minority, comprising less than 2% of the total population, compared to over 70% for small cells (≤4 µm). A comparable trend was reported by Chen et al. [49] in a *Auxenochlorella protothecoides* population, a species with a multiple-fission cell cycle similar to that of *C. zofingiensis*, where the proportion of mother cells decreased sharply from 8% on day 0 to only 1% by day 6 under photoautotrophic cultivation with white LED illumination.

During cultivation, the biovolume fraction of small cells (≤4 µm) decreased significantly, even though cell counts indicated that they remain the predominant population numerically. In contrast, larger cells (>12 µm) increased their contribution to the total biovolume, despite remaining a minority in terms of cell number, independent of light spectral composition or stress conditions. By comparison, Gao et al. [65] reported a decrease in *Chlorella vulgaris* cell biovolume over time during biofilm cultivation, likely resulting from light limitation due to steep gradients within the biofilm. In this study, however, cultures were maintained in suspension with thorough mixing, ensuring a more uniform light distribution. Moreover, Gao et al. [65] focused on cell counts, without reporting the relative biovolume of subpopulations, which limits direct comparison and underscores the value of biovolumetric analysis for a more comprehensive understanding of population dynamics.

Under N^−^ and N^−^S^+^ conditions, *C. zofingiensis* cultures exposed to red light exhibited pronounced population heterogeneity throughout the whole cultivation period, beginning as early as day 1. In contrast, under blue light, cells with diameters ≤ 4 µm dominated, contributing up to 50% of the total biovolume. Under green light, a broad spectrum of cell sizes was observed, with a gradual increase in the proportion of larger cells (≥16 µm) over time. These findings are consistent with those of Sánchez-Alvarez et al. [66], who reported that *Marinichlorella kaistiae* tended to maintain mother cells under nutrient-replete conditions, whereas daughter cells predominated under nutrient limitation. This highlights the influence of nutrient availability and light quality on the balance between cell growth and division. Nutrient depletion appeared to favor the formation of smaller cells, whose higher surface-to-biovolume ratio enhanced nutrient uptake efficiency, whereas larger cells possess greater nutrient storage capacity, allowing them to persist under limiting conditions. Consequently, distinct population structures emerged under different nutritional and light environments, reflecting adaptive strategies that balance nutrient acquisition, storage, and growth [67].

Under N^+^S^+^ conditions, cultures exposed to red and green light exhibited comparable population heterogeneity and cell size distributions, characterized by a progressive increase in the proportion of large cells (≥16 µm) over time—from approximately 20% on day 1 to around 65% and 60% on days 4 and 5, respectively. In contrast, cultures grown under blue light were dominated by small cells, with the biovolumetric fraction of large cells remaining below 20% on days 4 and 5. No significant differences were observed between the N^+^S^+^ and N^+^ conditions under blue light. However, under red and green light conditions, distinct responses emerged: under red light, the proportion of 20 µm cells was higher in N^+^S^+^ than in N^+^, whereas under green light, the fraction of large cells was lower in N^+^S^+^ compared to N^+^. These observations indicate that moderate osmotic stress interacts with light quality to shape *C. zofingiensis* population structure: it had minimal effect under blue light, promoted the accumulation of large cells under red light, and restricted the proportion of large cells under green light.

### 2.5. Multivariate Statistical Analysis of Subpopulations Structure

**Figure 4:**
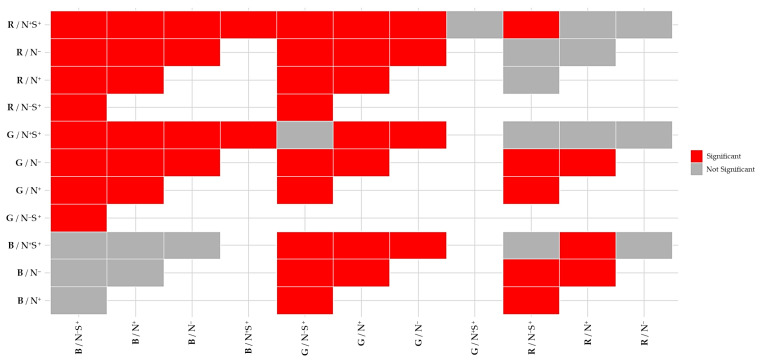
Matrix of pairwise PERMANOVA results for the different process conditions. Colors indicate the significance of pairwise multivariate differences after Benjamini–Hochberg false discovery rate (FDR) correction, with red denoting significant differences (FDR-adjusted *p* < 0.05) and grey indicating non-significant comparisons (FDR-adjusted *p* ≥ 0.05). The letters and symbols stand for different conditions, R for red light, B for blue light and G for green light. N^+^ (control); N^−^ (nitrate depleted); N^+^S^+^ (moderate salinity); N^−^S^+^ (nitrate depleted and moderate salinity).

Multivariate differences in cell diameter–class distributions among the different process conditions were assessed using PERMANOVA. The global PERMANOVA revealed a highly significant effect of treatment on subpopulation structure (*p* = 0.001), indicating that at least one condition differed in multivariate space. Post hoc, pairwise PERMANOVA tests were then conducted to identify the significant differences between the experimental treatments, with adjusted *p*-values calculated using the Benjamini–Hochberg false discovery rate procedure to account for multiple comparisons. The results of these pairwise analyses are summarized in Figure 4, which provides an overview of significant and non-significant differences among the process conditions.

The FDR-corrected pairwise PERMANOVA results (Figure 4) revealed clear, structured differentiation in the size-class distributions of the subpopulations, indicating that these distributions were strongly treatment-dependent. Most treatment combinations differed significantly from one another, as reflected by the prevalence of significant comparisons (red rectangles) across the matrix. This confirms that there has been substantial restructuring of the composition of the subpopulations in response to the applied process conditions.

A notable pattern emerged with regard to light-dependent responses. Significant differences were evident in treatments under red and green light, both within and across process conditions. This indicates a high sensitivity of population structure to changes in nitrogen availability and moderate salinity under these light spectral compositions. In contrast, several comparisons involving blue light, particularly between N^+^ and N^+^S^+^ conditions, were not significant. This suggests that moderate osmotic stress induces relatively minor changes in subpopulation structure under this light spectral composition.

Considered together with the analysis of subpopulation size distributions (Figure 3), these multivariate results revealed a clear hierarchy in light-dependent cell size responses. Green light most strongly promoted the appearance and accumulation of larger cell size classes, followed by red light. In contrast, blue light maintained a population structure dominated by small cells. This constrained size distribution under blue light was consistent with the limited number of significant differences observed across process conditions for this light spectral composition. This indicates that population structure was less sensitive to nutrient availability and moderate osmotic stress under blue light than under red and green light treatments.

These observed patterns collectively demonstrated that process conditions strongly and significantly influenced subpopulation dynamics and structure in *C. zofingiensis*. The marked differences across experimental treatments emphasize the importance of considering a combination of operational parameters, such as light quality and stress conditions, when analyzing and optimizing *C. zofingiensis* cultures.

## 3. Materials and Methods

### 3.1. Microalgal Strain

The green microalga *C. zofingiensis* (SAG 211–14, Göttingen, Germany) was obtained from the Culture Collection of Algae at Göttingen University. The strain was pre-cultured in 150 mL HD100 cultivator (CellDEG GmbH, Berlin, Germany) with bubble-free aerated CO_2_-enriched air (3% *v*/*v*) at room temperature (22 ± 2 °C) and an incident light intensity of 150 µmol∙m^−2^∙s^−1^, with the spectral composition of the light source provided in the Supplementary Materials. An industrial growth medium composed of 0.5 g∙L^−1^ NPK fertilizer (Hauert MANNA Düngerwerke GmbH, Nürnberg, Germany), supplemented by 0.5 g∙L^−1^ NaNO_3_ was used for the preculture.

### 3.2. Experimental Design and Culture Conditions

Precultured cells at the exponential growth phase were washed once with a 0.9% NaCl solution, then diluted with fresh medium to an optical density OD_750nm_ of 1 (i.e., 0.62 mg∙mL^−1^ of biomass dry weight concentration). To explore the population dynamics of *C. zofingiensis* at the different process conditions, cultures cultivated under three different light spectral compositions (red, blue, and green), provided by a LED panel (LED-KE 308, DH Licht GmbH, Wülfrath, Germany), and four conditions: control (N^+^) with 1 g∙L^−1^ NaNO_3_, nitrogen starvation only (N^−^) with 15 mg∙L^−1^ NaNO_3_, moderate osmotic stress only (N^+^S^+^) with 11.68 g∙L^−1^ NaCl (0.2 M) and nitrogen starvation combined to moderate osmotic stress (N^−^S^+^) with 1 g∙L^−1^ NaNO_3_ and 11.68 g∙L^−1^ NaCl. The selected salinity and nitrogen depletion levels were determined based on preliminary experiments, which identified conditions that induced physiological stress while maintaining active growth. Each condition was conducted in duplicates, with 150 mL working volume in HD100 cultivators (CellDEG GmbH, Berlin, Germany) with bubble-free aerated CO_2_-enriched air (3% *v*/*v*) at room temperature (22 ± 2 °C) and an incident light intensity of 150 µmol∙m^−2^∙s^−1^ for each light spectral composition.

### 3.3. Flow Cytometry Analysis

All samples were processed using the CyFlow Cube 8 (Sysmex Deutschland GmbH, Norderstedt, Germany), following the procedures as described in previous work by Ihadjadene et al. [18]. Briefly, samples from the cultivators were diluted in a 0.9% NaCl solution to a cell density of 7.5 × 10^6^–8 × 10^6^ cells/mL (OD_750nm_ approx. 0.03). Microalgal cells were gated using a two-dimensional FSC versus FL3 dot plot, where FL3 corresponds to chlorophyll autofluorescence of the microalgae cells. This gate was applied to isolate the microalgal population to exclude debris and electronic noise. Subsequently, FSC histograms were generated from the gated microalgal population only and distinct cell diameter classes were determined by applying size thresholds to the FSC distributions according to the established bead-based calibration (see Section 3.4 Microalgal Cell Size Determination).

All FCM data were processed using FlowJo software version 10.9.0 (BD Life Sciences, Ashland, OR, USA). Gating of microalgae populations was done in the two-dimensional FSC vs. FL3 dot plot in order to remove the background noise.

### 3.4. Microalgal Cell Size Determination

Counting microalgae cells and measuring their size using traditional methods as light microscopy is a very tedious and time-consuming task, allowing analyzing only a few tens to hundreds of cells [12,49]. FCM is a powerful high-throughput method allowing microalgae cell counting up to 10,000 cell/s, however, estimating their size presents a challenge, as FSC values are inherently qualitative and reported in arbitrary units to cell’s cross section, rather than standardized measurements such as micrometers (µm) [48]. To address this, a suspension of a mixture of spherical reference microbeads (Sysmex Deutschland GmbH, Norderstedt, Germany), with defined diameters of 1 µm, 3 µm, and 10 µm was used for particle size calibration. The spherical shape of the beads was chosen as it matches the morphology of *C. zofingiensis* cells. This approach enabled the transformation of FSC signals into standardized units, allowing for accurate size determination of the cells.

### 3.5. Light Microscopy

**Figure 5:**
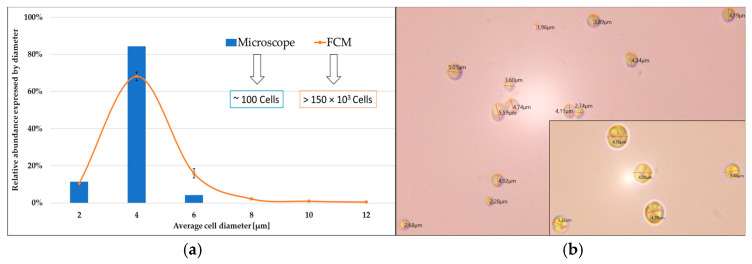
Comparison between FCM and light microscopy results of cell size distribution (**a**). Light microscopy images for cell size determination (**b**).

To validate the accuracy of cell size measurements obtained by FCM, cell diameters were also determined using microscopy (Zeiss Axiostar Plus, Carl Zeiss Microscopy Deutschland GmbH, Oberkochen, Germany) equipped with a MikroCam SP 5.0 MP camera (Bresser GmbH, Rhede, Germany). The average cell size measured microscopically was consistent with the values obtained by FCM (Figure 5).

Additionally, light microscopy was employed to check for potential contamination in the cultures throughout the experiments.

### 3.6. Cell Size Class Definition

Cell size distributions were quantified by assigning individual cells to discrete diameter classes based on their measured equivalent spherical diameter. Size classes were defined as non-overlapping diameter intervals centered on nominal class values (2, 4, 6, 8, 10, 12, 16 and 20 µm), selected because they correspond to the dominant cell sizes observed in the raw diameter distributions, with the majority of cells within each class clustering around the corresponding nominal value. Specifically, cells with diameters between 1.8–3.0 µm were assigned to the 2 µm class, 3.0–5.0 µm to the 4 µm class, 5.0–7.0 µm to the 6 µm class, 7.0–9.0 µm to the 8 µm class, 9.0–11.0 µm to the 10 µm class, 11.0–14.0 µm to the 12 µm class, 14.0–18.0 µm to the 16 µm class, and cells larger than 18.0 µm to the 20 µm class. Defining size classes centered on these dominant diameters therefore allowed the main subpopulations present in the cultures to be captured accurately, while avoiding arbitrary thresholds or excessive fragmentation of the size distribution.

### 3.7. Cell Biovolume

Based on microscopic observations, the cells of *C. zofingiensis* are round-shaped; thus, their biovolume can be determined according to their measured diameter as follows:

(2) $$V=\frac{4}{3}\cdot A\sqrt{\frac{A}{\pi }}$$

(3) $$A=\pi \cdot {\left(\frac{d}{2}\right)}^{2}$$

where *d* is the diameter of algae cells in (µm) and *A* is their projection area in (µm^2^).

### 3.8. Statistical Analysis

To assess multivariate differences in cell-size class distributions among the process conditions, permutational multivariate analysis of variance (PERMANOVA) was applied to the compositional dataset. Prior to analysis, all proportional diameter-class data were first transformed using a centered log–ratio (CLR) transformation to account for compositional constraints. When a significant global effect was detected, pairwise PERMANOVA comparisons were conducted between all Light × Condition combinations. *p*-values from pairwise tests were adjusted using the Benjamini–Hochberg false discovery rate (FDR) correction to account for multiple comparisons. All analyses were performed in RStudio version 2024.12.1 Build 563 (Posit Software, PBC, Boston, MA, USA).

## 4. Conclusions

*Chromochloris zofingiensis* subpopulation dynamics are strongly shaped by the combined effects of nutrient availability, moderate osmotic stress, and light quality. While small cells dominate numerically, larger cells contribute disproportionately to total biovolume and biomass. Red light promotes growth and large-cell accumulation, blue light maintains populations dominated by small cells, and green light produces intermediate heterogeneity. Biovolume-based analyses provide critical insights into biomass distribution and metabolic potential, linking cell morphology to productivity, by integrating biochemical measurements at the subpopulation level—such as pigment content, lipid accumulation, or other intracellular metabolites—would further enhance the biological and functional relevance of the proposed biovolume-based metrics. In particular, quantifying variations in biosynthetic performance by measuring metabolite concentrations in distinct cell subpopulations isolated using fluorescence-activated cell sorting (FACS) would enable a direct linkage between cell size, physiological state, and metabolic activity [16,68].

These findings offer a framework for optimizing phototrophic cultivation strategies to enhance growth and metabolite yield, while also providing a foundation for advanced predictive modeling approaches—such as population balance models—that capture population heterogeneity. By quantitatively describing cell-to-cell variability and different phases of the cellular life cycle, these models can significantly improve the design, monitoring, and control of microalgae cultivation and processing across the biorefinery process chain.

## Figures and Tables

**Table 1:** Detailed comparison of *C. zofingiensis* subpopulation composition on day 4 based on cell diameter versus cell biovolume (Figure 2), for the N^+^S^+^ condition under red light illumination.

Cell Diameter [µm]	Cell Number [Cell/mL]	Relative Cell Abundance [%]	Relative Biovolumetric Fraction [%]
2	3.90 × 10^6^ ± 1.22 × 10^5^	11	1
4	22.88 × 10^6^ ± 3.68 × 10^5^	65	11.5
6	5.43 × 10^6^ ± 2.70 × 10^5^	15.5	9
8	8.66 × 10^5^ ± 4.97 × 10^4^	2.5	3
10	3.24 × 10^5^ ± 1.09 × 10^4^	1	2
12	3.31 × 10^5^ ± 1.35 × 10^4^	1	4
16	4.51 × 10^5^ ± 2.48 × 10^4^	1.3	14
20	9.03 × 10^5^ ± 3.04 × 10^4^	2.5	55

## Data Availability

Data will be made available on request.

## Supplementary Materials

The following supporting information can be downloaded at: https://www.mdpi.com/article/10.3390/plants15050724/s1.

## References

[1] Chisti Y., Jacob-Lopes E., Maroneze M.M., Queiroz M.I., Zepka L.Q. (2020). Chapter 1—Microalgae Biotechnology: A Brief Introduction. Handbook of Microalgae-Based Processes and Products.

[2] Yu B.S., Pyo S., Lee J., Han K. (2024). Microalgae: A Multifaceted Catalyst for Sustainable Solutions in Renewable Energy, Food Security, and Environmental Management. Microb. Cell Fact..

[3] Bañuelos-Hernández B., Beltrán-López J.I., Rosales-Mendoza S., Kim S.-K. (2015). Chapter 18—Production of Biopharmaceuticals in Microalgae. Handbook of Marine Microalgae.

[4] Couteau C., Coiffard L., Levine I.A., Fleurence J. (2018). Chapter 15—Microalgal Application in Cosmetics. Microalgae in Health and Disease Prevention.

[5] Dewi I.C., Falaise C., Hellio C., Bourgougnon N., Mouget J.-L., Levine I.A., Fleurence J. (2018). Chapter 12—Anticancer, Antiviral, Antibacterial, and Antifungal Properties in Microalgae. Microalgae in Health and Disease Prevention.

[6] Goiris K., Muylaert K., De Cooman L., Kim S.-K. (2015). Chapter 17—Microalgae as a Novel Source of Antioxidants for Nutritional Applications. Handbook of Marine Microalgae.

[7] Kaur S., Khattar J.I.S., Singh Y., Khattar J.I.S., Singh D.P., Singh R.P. (2025). Potential of Algal Metabolites in Cosmetics and Personal Care Products. Industrial and Biotechnological Applications of Algae.

[8] Mendes M.C., Navalho S., Ferreira A., Paulino C., Figueiredo D., Silva D., Gao F., Gama F., Bombo G., Jacinto R. (2022). Algae as Food in Europe: An Overview of Species Diversity and Their Application. Foods.

[9] Morançais M., Mouget J.-L., Dumay J., Levine I.A., Fleurence J. (2018). Chapter 7—Proteins and Pigments. Microalgae in Health and Disease Prevention.

[10] Ng D.H.P., Ng Y.K., Shen H., Lee Y.K., Kim S.-K. (2015). Chapter 6—Microalgal Biotechnology: The Way Forward. Handbook of Marine Microalgae.

[11] Ryu B., Himaya S.W.A., Kim S.-K., Kim S.-K. (2015). Chapter 20—Applications of Microalgae-Derived Active Ingredients as Cosmeceuticals. Handbook of Marine Microalgae.

[12] Hyka P., Lickova S., Přibyl P., Melzoch K., Kovar K. (2013). Flow Cytometry for the Development of Biotechnological Processes with Microalgae. Biotechnol. Adv..

[13] Akimov A.I., Solomonova E.S., Shoman N.Y. (2024). Estimation Physiological State and Carotenoid Content of *Dunaliella salina* (Teod.) Using Flow Cytometry and Variable Fluorescence Methods. Aquacult Int..

[14] Benner P., Meier L., Pfeffer A., Krüger K., Oropeza Vargas J.E., Weuster-Botz D. (2022). Lab-Scale Photobioreactor Systems: Principles, Applications, and Scalability. Bioprocess Biosyst. Eng..

[15] Obidi P.O., Bayless D.J. (2025). Mechanical Characterization of Algal Cultivation Systems for Enhanced Mass Transfer. Algal Res..

[16] Delvigne F., Zune Q., Lara A.R., Al-Soud W., Sørensen S.J. (2014). Metabolic Variability in Bioprocessing: Implications of Microbial Phenotypic Heterogeneity. Trends Biotechnol..

[17] Usai A., Theodoropoulos C., Di Caprio F., Altimari P., Cao G., Concas A. (2023). Structured Population Balances to Support Microalgae-Based Processes: Review of the State-of-Art and Perspectives Analysis. Comput. Struct. Biotechnol. J..

[18] Ihadjadene Y., Walther T., Krujatz F. (2022). Optimized Protocol for Microalgae DNA Staining with SYTO9/SYBR Green I, Based on Flow Cytometry and RSM Methodology: Experimental Design, Impacts and Validation. Methods Protoc..

[19] Krujatz F., Lode A., Brüggemeier S., Schütz K., Kramer J., Bley T., Gelinsky M., Weber J. (2015). Green Bioprinting: Viability and Growth Analysis of Microalgae Immobilized in 3D-Plotted Hydrogels versus Suspension Cultures. Eng. Life Sci..

[20] López-Gálvez J., Schiessl K., Besmer M.D., Bruckmann C., Harms H., Müller S. (2023). Development of an Automated Online Flow Cytometry Method to Quantify Cell Density and Fingerprint Bacterial Communities. Cells.

[21] Park K.-H., Jho E.H., Hwang S.-J. (2023). Quantitative Viability Assessment of Microalgae for Advanced Wastewater Treatment by Flow Cytometry. KSCE J. Civ. Eng..

[22] Song Y., Lee Y. (2024). Brief Guide to Flow Cytometry. Mol. Cells.

[23] Bleisch R., Ihadjadene Y., Torrisi A., Walther T., Mühlstädt G., Steingröwer J., Streif S., Krujatz F. (2025). Physiological Adaptation of *Chromochloris zofingiensis* in Three-Phased Cultivation Performed in a Pilot-Scale Photobioreactor. Life.

[24] Gorgich M., Martins A.A., Mata T.M., Caetano N.S. (2021). Composition, Cultivation and Potential Applications of *Chlorella zofingiensis*—A Comprehensive Review. Algal Res..

[25] Liu J., Sun Z., Gerken H., Liu Z., Jiang Y., Chen F. (2014). *Chlorella zofingiensis* as an Alternative Microalgal Producer of Astaxanthin: Biology and Industrial Potential. Mar. Drugs.

[26] Wood E.E., Ross M.E., Jubeau S., Montalescot V., Stanley M.S. (2022). Progress towards a Targeted Biorefinery of *Chromochloris zofingiensis*: A Review. Biomass Conv. Bioref..

[27] Zhang Y., Ye Y., Bai F., Liu J. (2021). The Oleaginous Astaxanthin-Producing Alga *Chromochloris zofingiensis*: Potential from Production to an Emerging Model for Studying Lipid Metabolism and Carotenogenesis. Biotechnol. Biofuels.

[28] Koren I., Boussiba S., Khozin-Goldberg I., Zarka A. (2021). *Chromochloris zofingiensis* (Chlorophyceae) Divides by Consecutive Multiple Fission Cell-Cycle under Batch and Continuous Cultivation. Biology.

[29] Bišová K., Zachleder V. (2014). Cell-Cycle Regulation in Green Algae Dividing by Multiple Fission. J. Exp. Bot..

[30] Ivanov I.N., Vítová M., Bišová K. (2019). Growth and the Cell Cycle in Green Algae Dividing by Multiple Fission. Folia Microbiol..

[31] Chowdhary A.K., Kishi M., Toda T. (2025). Astaxanthin Induction in *Chromochloris zofingiensis* by Transition of Nutritional Modes. J. Appl. Phycol..

[32] Ihadjadene Y., Ascoli L., Syed T., Urbas L., Walther T., Mühlstädt G., Streif S., Krujatz F. (2025). Experimental and Model-Based Parameterization of the Fundamental Process Kinetics of *Chromochloris zofingiensis*. Algal Res..

[33] Kou Y., Liu M., Sun P., Dong Z., Liu J. (2020). High Light Boosts Salinity Stress-Induced Biosynthesis of Astaxanthin and Lipids in the Green Alga *Chromochloris zofingiensis*. Algal Res..

[34] Ma R., Ma X., Qiao Y., Wang B., Ho S.-H., Chen J., Xie Y. (2025). Improved Production of Astaxanthin in Heterotrophic *Chromochloris zofingiensis* through Optimized Culture Conditions Incorporating an Efficient Fed-Batch Strategy. Algal Res..

[35] Minyuk G., Sidorov R., Solovchenko A. (2020). Effect of Nitrogen Source on the Growth, Lipid, and Valuable Carotenoid Production in the Green Microalga *Chromochloris zofingiensis*. J. Appl. Phycol..

[36] Sun H., Ren Y., Lao Y., Li X., Chen F. (2020). A Novel Fed-Batch Strategy Enhances Lipid and Astaxanthin Productivity without Compromising Biomass of *Chromochloris zofingiensis*. Bioresour. Technol..

[37] Wang Y., Zhao W., Wang J., Yang S., Liu J., Zheng J., Mou H., Sun H. (2025). Enhancing Astaxanthin Production from *Chromochloris zofingiensis* via Blue Light and Exogenous Inducers in Plate Photobioreactors. Bioresour. Technol..

[38] Wang Y., Sun H., Wang J., Gu Z., Chen F., Mou H., Yang S. (2022). A Mixotrophic Preculture Strategy Enhances Biomass and Astaxanthin Productivity of *Chromochloris zofingiensis*. https://link.springer.com/article/10.1007/s00253-023-12873-x.

[39] Ugwu C., Hashiguchi A., Nagare H. (2024). Influence of Different Light Wavelengths and Carbon Sources on Energy Utilization Efficiency by *Chromochloris zofingiensis* for Cell Growth and Carotenoid Synthesis. Bioresour. Technol. Rep..

[40] Maltsev Y., Maltseva K., Kulikovskiy M., Maltseva S. (2021). Influence of Light Conditions on Microalgae Growth and Content of Lipids, Carotenoids, and Fatty Acid Composition. Biology.

[41] Wang S.-K., Stiles A.R., Guo C., Liu C.-Z. (2014). Microalgae Cultivation in Photobioreactors: An Overview of Light Characteristics. Eng. Life Sci..

[42] Chen H.-B., Wu J.-Y., Wang C.-F., Fu C.-C., Shieh C.-J., Chen C.-I., Wang C.-Y., Liu Y.-C. (2010). Modeling on Chlorophyll a and Phycocyanin Production by *Spirulina platensis* under Various Light-Emitting Diodes. Biochem. Eng. J..

[43] Baba M., Kikuta F., Suzuki I., Watanabe M.M., Shiraiwa Y. (2012). Wavelength Specificity of Growth, Photosynthesis, and Hydrocarbon Production in the Oil-Producing Green Alga *Botryococcus braunii*. Bioresour. Technol..

[44] Chen J., Liu L., Wei D. (2017). Enhanced Production of Astaxanthin by *Chromochloris zofingiensis* in a Microplate-Based Culture System under High Light Irradiation. Bioresour. Technol..

[45] Mao X., Zhang Y., Wang X., Liu J. (2020). Novel Insights into Salinity-Induced Lipogenesis and Carotenogenesis in the Oleaginous Astaxanthin-Producing Alga *Chromochloris zofingiensis*: A Multi-Omics Study. Biotechnol. Biofuels.

[46] Liu J., Sun Z., Mao X., Gerken H., Wang X., Yang W. (2019). Multiomics Analysis Reveals a Distinct Mechanism of Oleaginousness in the Emerging Model Alga *Chromochloris zofingiensis*. Plant J..

[47] Ortolani C. (2022). Flow Cytometry Today: Everything You Need to Know About Flow Cytometry.

[48] Davies P., Cavallaro M., Hebenstreit D. (2025). Single-Calibration Cell Size Measurement With Flow Cytometry. Cytom. Part A.

[49] Chen H., Sosa A., Chen F. (2024). Growth and Cell Size of Microalga *Auxenochlorella protothecoides* AS-1 under Different Trophic Modes. Microorganisms.

[50] Dunker S., Boyd M., Durka W., Erler S., Harpole W.S., Henning S., Herzschuh U., Hornick T., Knight T., Lips S. (2022). The Potential of Multispectral Imaging Flow Cytometry for Environmental Monitoring. Cytom. Part A.

[51] Gincley B., Khan F., Alam M.M., Hartnett E., Kim G.-Y., Molitor H.R., Fisher A., Bradley I., Guest J., Pinto A.J. (2025). Morphotype-Resolved Characterization of Microalgal Communities in a Nutrient Recovery Process with ARTiMiS Flow Imaging Microscopy. Water Res..

[52] Işıl Ç., de Haan K., Göröcs Z., Koydemir H.C., Peterman S., Baum D., Song F., Skandakumar T., Gumustekin E., Ozcan A. (2021). Phenotypic Analysis of Microalgae Populations Using Label-Free Imaging Flow Cytometry and Deep Learning. ACS Photonics.

[53] Helbig C., Menzen T., Wuchner K., Hawe A. (2022). Imaging Flow Cytometry for Sizing and Counting of Subvisible Particles in Biotherapeutics. J. Pharm. Sci..

[54] Lee P.-Y., Pahija E., Liang Y.-Z., Yeoh K.-P., Hui C.-W., Friedl A., Klemeš J.J., Radl S., Varbanov P.S., Wallek T. (2018). Population Balance Equation Applied to Microalgae Harvesting. Computer Aided Chemical Engineering.

[55] Lee P.Y., Yeoh K.P., Hui C.W., Kiss A.A., Zondervan E., Lakerveld R., Özkan L. (2019). Population Balance Equation Applied to Microalgae Filtration. Computer Aided Chemical Engineering.

[56] Pahija E., Liang Y., Hui C.W., Kravanja Z., Bogataj M. (2016). Population Balance Applied to Microalgae Growth. Computer Aided Chemical Engineering.

[57] Pahija E., Lee P.Y., Hui C.-W., Sin G. (2022). Modelling of Harvesting Techniques for the Evaluation of the Density of Microalgae. Appl. Biochem. Biotechnol..

[58] Finkel Z.V. (2001). Light Absorption and Size Scaling of Light-Limited Metabolism in Marine Diatoms. Limnol. Oceanogr..

[59] Marañón E. (2015). Cell Size as a Key Determinant of Phytoplankton Metabolism and Community Structure. Annu. Rev. Mar. Sci..

[60] Mcnair H., Hammond C.N., Menden-Deuer S. (2021). Phytoplankton Carbon and Nitrogen Biomass Estimates Are Robust to Volume Measurement Method and Growth Environment. J. Plankton Res..

[61] Schagerl M., Siedler R., Konopáčová E., Ali S.S. (2022). Estimating Biomass and Vitality of Microalgae for Monitoring Cultures: A Roadmap for Reliable Measurements. Cells.

[62] Minyuk G., Chelebieva E., Chubchikova I., Dantsyuk N., Drobetskaya I., Sakhon E., Chekanov K., Solovchenko A., Minyuk G., Chelebieva E. (2017). Stress-Induced Secondary carotenogenesis in *Coelastrella rubescens* (Scenedesmaceae, Chlorophyta), a Producer of Value-Added Keto-Carotenoids. Algae.

[63] Finkel Z.V., Follows M.J., Irwin A.J. (2016). Size-Scaling of Macromolecules and Chemical Energy Content in the Eukaryotic Microalgae. J. Plankton Res..

[64] Pahija E., Hui C.-W. (2021). A Practical Approach for Modelling the Growth of Microalgae with Population Balance Equation. New Biotechnol..

[65] Gao Y., Bernard O., Fanesi A., Perré P., Lopes F. (2024). The Effect of Light Intensity on Microalgae Biofilm Structures and Physiology under Continuous Illumination. Sci. Rep..

[66] Sánchez-Alvarez E.L., González-Ledezma G., Bolaños Prats J.A., Stephano-Hornedo J.L., Hildebrand M. (2017). Evaluating *Marinichlorella kaistiae* KAS603 Cell Size Variation, Growth and TAG Accumulation Resulting from Rapid Adaptation to Highly Diverse Trophic and Salinity Cultivation Regimes. Algal Res..

[67] Andersen K.H., Visser A.W. (2023). From Cell Size and First Principles to Structure and Function of Unicellular Plankton Communities. Prog. Oceanogr..

[68] Xiao Y., Bowen C.H., Liu D., Zhang F. (2016). Exploiting Nongenetic Cell-to-Cell Variation for Enhanced Biosynthesis. Nat. Chem. Biol..

