# Supplementary material for: Population Dynamics Analysis of Chromochloris zofingiensis: A Flow-Cytometry-Based Approach
Source: Plants (Basel). 2026 Feb 27;15(5):724. doi: 10.3390/plants15050724 (PMC12986623; doi:10.3390/plants15050724)
Supplement: Supplementary file 1 [file plants-15-00724-s001.zip › plants-4088023-supplementary.pdf]

Supplementary Material

for

Population Dynamics Analysis of *Chromochloris zofingiensis*: A Flow Cytometry-Based Approach

Yob Ihadjadene [1,2,3], Alina Wulff [4], Thomas Walther [2], Stefan Streif [1,5] and Felix Krujatz [1,3] *

[1] Professorship Automatic Control & System Dynamics, Chemnitz University of Technology, 09126 Chemnitz, Germany; yob.ihadjadene@etit.tu-chemnitz.de (Y.I.); stefan. streif@ etit.tu-chemnitz.de (S.S.); felix.krujatz@etit.tu-chemnitz.de (F.K.)
[2] Institute of Natural Materials Technology, Dresden University of Technology, 01069 Dresden, Germany; thomas_walther@tu-dresden.de (T.W.)
[3] biotopa gGmbH—Center for Applied Aquaculture & Bioeconomy, 01454 Radeberg, Germany; info@biotopa.org (F.K.)
[4] Department of Biotechnology, Technische Hochschule Mannheim, 68163 Mannheim; alina.wulff1@stud.hs-mannheim.de (A.W.)
[5] Fraunhofer Institute for Molecular Biology and Applied Ecology, Department of Bioresources, 35392 Giessen, Germany; stefan.streif@ime.fraunhofer.de (S.S.)
* Correspondence: felix.krujatz@etit.tu-chemnitz.de (F.K.)

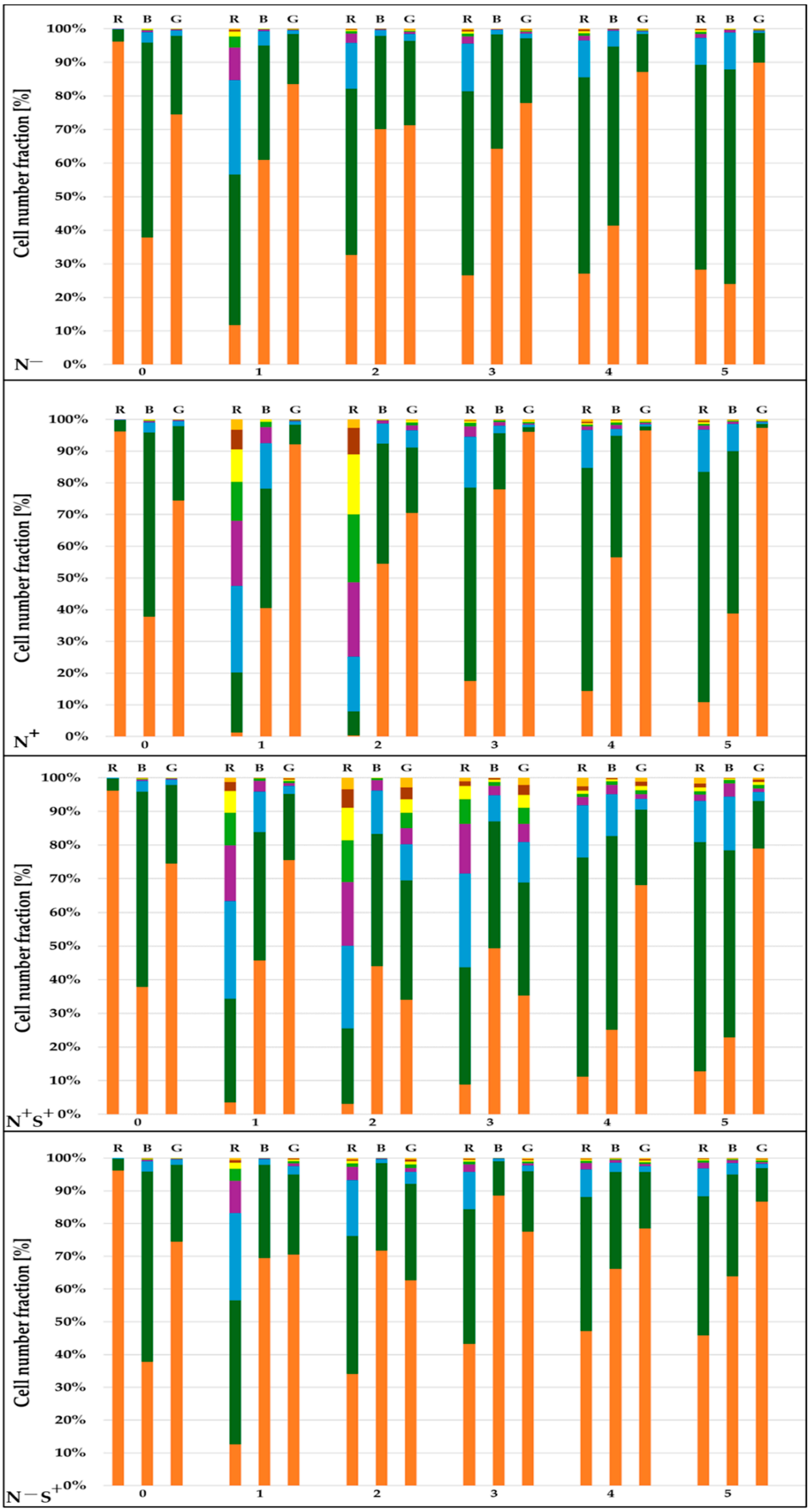

Fig. S1: Temporal evolution of the relative cell number distribution of *C. zofingiensis* cell diameter classes. Colors indicate distinct cell diameter classes, with the relative cell number fraction (%) of each class normalized to the total population (y-axis). Light conditions are denoted by R (red), B (blue), and G (green). Nutrient and osmotic stress conditions are defined as follows: N+ (control); N⁻ (nitrate depleted); N+S+ (moderate salinity); N⁻S+ (nitrate depleted and moderate salinity).

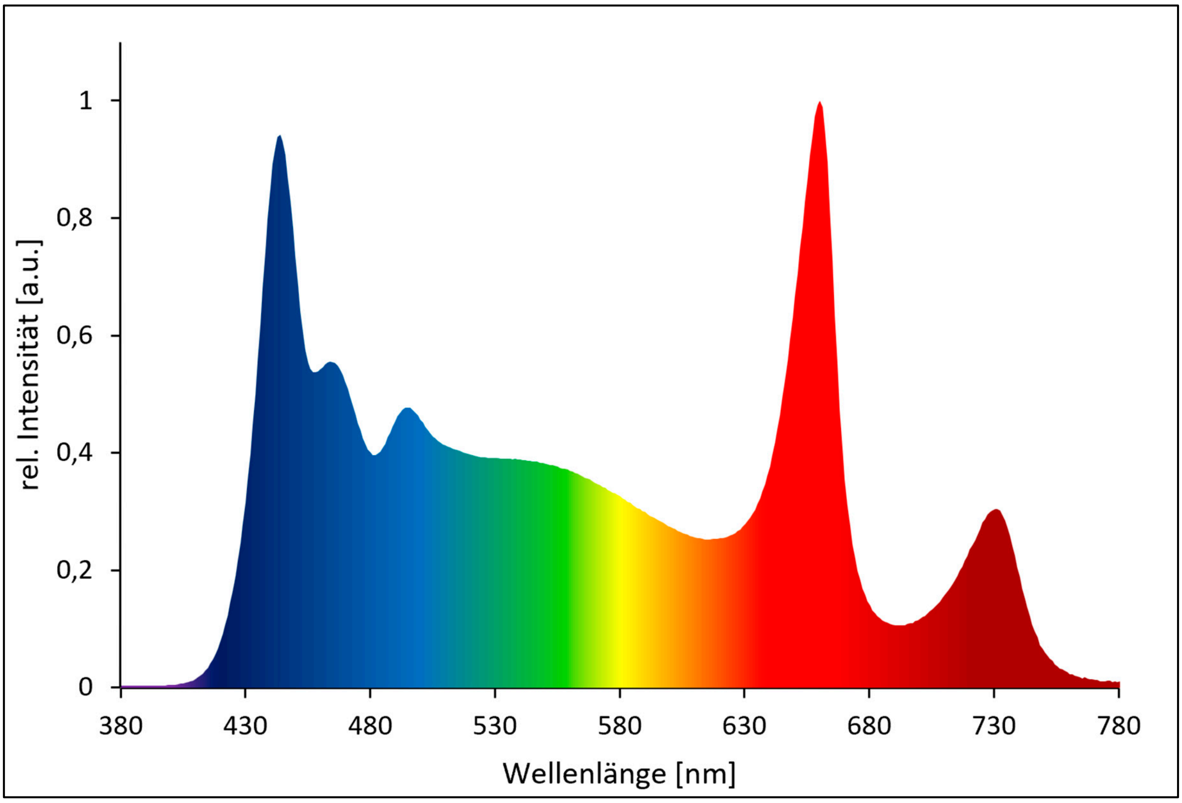

Fig. S2: Spectral composition of the light source used for culture illumination.
